# Free transverse vibration analysis of Non-uniform Nano-Timoshenko beams rotating around two perpendicular axes using differential quadrature method

**DOI:** 10.1038/s41598-026-54986-z

**Published:** 2026-05-25

**Authors:** Ali M. Mohsen, Rashid Mjheid Fadwi, Banaz Shahab Haji, Narinderjit Singh Sawaran Singh, Zahraa Abed Hussein, Salah S. Hamd, Ali Fotohi, Ahmad Keshavarzi, Soheil Salahshour

**Affiliations:** 1 Advanced Technical College, University of Warith Al-Anbiyaa, Karbala, Iraq; 2 Department of Medical Instruments Engineering Techniques, College of Engineering, University of Al Maarif, Al Anbar, 31001 Iraq; 3 Medical Radiology Imaging Department, College of Health Sciences, Lebanese French University (LFU), Erbil, Kurdistan Region Iraq; 4 Faculty of Data Science and Information Technology, INTI International University, Persiaran Perdana BBN, Putra Nilai, Nilai, 71800 Malaysia; 5 University of Manara, Amarah, Maysan Iraq; 6 Department of Mechanical Engineering, Kho.C., Islamic Azad University, Khomeinishahr, Iran; 7 Faculty of Engineering and Natural Sciences, Istanbul Okan University, Istanbul, Turkey; 8 Faculty of Engineering and Natural Sciences, Bahcesehir University, Istanbul, Turkey; 9 Research Center of Applied Mathematics, Khazar University, Baku, Azerbaijan; 10 Faculty of Science and Letters, Piri Reis University, Tuzla, Istanbul, Turkey

**Keywords:** Nano-rotor, Timoshenko beam theory, Eringen’s non-local elasticity theory, Differential quadrature method Process innovation, Engineering, Nanoscience and technology, Physics

## Abstract

This research examined the vibrational characteristics of nano-rotors using Timoshenko beam theory, Eringen’s non-local elasticity theory, and gyroscopic effects. The governing equations and boundary conditions for whirling analysis were formulated for a nano-rotor with a non-uniform cross-section revolving around two orthogonal axes. The equations were converted to a non-dimensional form by introducing dimensionless parameters and then solved numerically using the differential quadrature technique. Numerical results validated the precision and convergence of the proposed solution. The findings indicate that, in the absence of rotation, the forward and backward vibration frequencies of the nano-rotor were similar. Nevertheless, once the rotor began rotating along its longitudinal axis, this symmetry was compromised. The forward frequencies increased, whilst the backward frequencies decreased. The frequency divergence became more pronounced with increasing rotational velocity, leading to higher critical velocities. Besides rotational effects, structural characteristics considerably influenced the vibrational response. Augmenting the shaft radius elevated both forward and backward frequencies, as well as critical velocities, illustrating that geometric alterations may be used to optimize rotor dynamics. Moreover, non-local elasticity increased the scale-dependence of phenomena. Increasing non-local parameters marginally increased the frequencies of the first vibration mode, while substantially decreasing those of higher modes, highlighting the nanoscale dependence of vibrational properties. The research indicated that the changes in the rotor’s diameter profile significantly affected its vibrational characteristics. A more pronounced drop in diameter along the rotor’s length increased first-mode frequencies and critical velocities, while diminishing those of higher modes. A greater rotor diameter increased the frequency difference between forward and backward modes, underscoring the significance of geometric design in regulating vibrational properties.

## Introduction

The natural frequencies, critical velocities (Vs), and mode shapes of a rotating shaft are crucial for rotor design and forced-vibration analysis. Practical utilizations of rotor dynamics include rotating shafts, turbines, and aeronautical equipment. Static beam models are inadequate for representing rotating shafts; therefore, it is preferable to advance the model to include rotor vibration analysis. The beam theory is incapable of modeling gyroscopic phenomena and distinguishing between stationary and rotating beams because it excludes shear deformation and rotational inertia^1–4^. Therefore, Timoshenko beam theory is preferable for modeling rotors^5,6^. Since the discovery of carbon nanotubes by Iijima^5^, nanomaterials have attracted significant interest from engineers and scientists. Currently, several nanostructures are being constructed and used as foundational elements in the emerging domain of nanotechnology. Numerous nanodevices integrate structural components such as beams and plates at the nanoscale. Given that the dimensions of these structures are diminutive, on the order of molecular distances, size effects are crucial for analyzing their mechanical behavior. The atomic and molecular modes need substantial computing resources; hence, simplified continuum mechanics theories that account for microscopic length scales serve as valuable tools for analyzing such structures. Eringen’s theory of non-local continuum mechanics was widely adopted among size-dependent continuum theories to explain phenomena such as wave propagation, dislocations, and fractures in nanostructures^7–10^. In non-local theory, small-scale effects were represented by positing that the stress at a given point depends on the strains at all points within the domain. This theory accounted for long-range interatomic interactions, yielding results akin to those of discrete-atomistic or molecular-dynamics simulations^11–13^. Researchers showed that circularly polarized light may induce rotation in nanotubes, thus prompting significant investigation into carbon nanotubes (CNTs) and nano-rotors in rotational dynamics. Pradhan et al.^14^ used non-local elasticity (NE) theory to examine the flap-wise bending vibration of a rotating uniform nanocantilever represented by an Euler–Bernoulli beam. They used the differential quadrature approach to address the issue. The vibration properties of the identical issues under initial pre-stress were investigated by Murmu et al.^15^. Applying a non-local Euler-Bernoulli beam, Narendar et al.^16^ examined the wave dispersion behavior of a spinning uniform nanotube. They considered the axial force as the maximum centrifugal force (at the nanocantilever’s root).

Farhadipour et al.^17^ employed the Euler-Bernoulli theory on a Pasternak foundation to simulate wave propagation in cantilever spinning nano-rotors, using the non-local theory of elasticity. Hamilton’s principle is utilized to derive the governing equations while accounting for non-local effects. Important results show how factors such as shear stiffness, rotational V, and the non-local scale affect wave dispersion properties. Interestingly, at high rotational Vs, flexural waves showed non-dispersive behavior, suggesting important ramifications for nanoscale structure design and analysis. Nonlinear wave dispersion in a nanodimension sandwich pipe with a bidirectional functionally graded core and piezoelectric devices was investigated by Guo et al.^18^. Hamilton’s principle was utilized to derive the governing equations, and COMSOL simulations were used to verify the findings. With an emphasis on the effects of applied voltage and piezoelectric patch position on phase V, an artificial neural network (ANN) was used for predictive modeling, offering insights for applications in the nanoindustry.

Afshari et al. provided a precise solution for examining the spinning behavior of rotors bearing concentrated weights^19^. They investigated the effects of the location of concentrated mass, translational inertia, and spin V on forward and backward frequencies. The findings showed that these factors had a considerable impact on rotor vibration characteristics, with their effects varying with position and inertia. Notably, the suggested technique achieved excellent efficiency, enabling the straightforward solution of a 2nd-order determinant to find the natural frequencies of rotors with any number of concentrated masses. Afshari et al.^20^ examined the free transverse vibration of multi-stepped rotors supported by numerous bearings using the differential quadrature element approach. They designed each bearing as four springs and used Timoshenko beam theory to account for gyroscopic effects. Differential quadrature rules were used in the study to develop the governing equations and boundary conditions. They used a Campbell diagram to analyze natural frequency fluctuations in spin angular V after confirming the procedure’s correctness and effectiveness. The results show that the suggested approach outperformed conventional analytical techniques in terms of efficiency and time, especially for rotors with multiple steps and bearings. The vibrations of a composite Timoshenko rotor were examined by Rahagi et al.^21^, accounting for both transverse and longitudinal-torsional vibrations. The governing equations and boundary conditions were derived using Hamilton’s principle, and the natural frequencies and mode shapes were ascertained using the differential quadrature technique. Additionally, they found two crucial Vs: the instability V, at which the first natural frequency declined to zero and causes instability, and the resonance V, at which the rotor spins close to a natural frequency.

In practical engineering applications, nanoscale rotating components, such as nanoturbines, nanactuators, and micro- and nano-electromechanical systems (MEMS/NEMS), often operate at high rotational speeds and under complex loading conditions, where accurate prediction of vibration characteristics, critical Vs, and dynamic stability is essential for reliable performance and failure prevention. At these scales, classical continuum theories were no longer sufficient because they lacked intrinsic length-scale parameters. To overcome this limitation, various size-dependent theories have been developed. Eringen’s NE theory captured long-range interatomic interactions using a single material length-scale parameter and provided an efficient framework for nanoscale analysis. However, more advanced models, such as the non-local strain gradient theory and the modified non-local strain gradient theory, introduced additional length-scale parameters, allowing the simultaneous representation of stiffness softening and hardening mechanisms. Recent studies, including those by Mirfatah et al.^22^, Vinh^23^, and Nosrati et al.^24^, showed that incorporating strain gradient effects can improve the accuracy of vibration predictions, particularly in complex geometries and auxetic structures, while review works, such as Garg et al.^25^, highlighted the integration of these higher-order theories within modern computational frameworks. Despite their improved accuracy, these theories typically involved increased mathematical complexity and computational cost. Therefore, the present study adopted Eringen’s NE theory as a balanced and efficient approach to capture essential size-dependent effects in the vibration analysis of rotating nano-rotors. Building upon the existing literature, this research provided a comprehensive analysis of the vibrational behavior of non-uniform nano-rotors by simultaneously incorporating non-local elasticity, geometric variations, and dual rotational effects. Unlike previous studies that primarily focused on isolated aspects, such as gyroscopic effects or functionally graded materials, the current work considered a broader range of design parameters, including shaft radius and diameter gradient, to systematically evaluate their impact on forward and backward whirling frequencies and critical Vs. The results demonstrate that geometric non-uniformity played a key role in governing frequency divergence and dynamic stability, while NE introduced distinct size-dependent behavior across different vibration modes. Furthermore, by formulating the governing equations in a non-dimensional framework and solving them using the differential quadrature method (DQM), the study offered an efficient and accurate strategy for capturing the coupled effects of rotation and nanoscale mechanics. This integrated modeling approach provided new insights into the design and optimization of nano-rotors and contributes to a deeper understanding of their dynamic performance in advanced nanoscale applications.

## Simulation details

### Governing equations

**Fig. 1:**
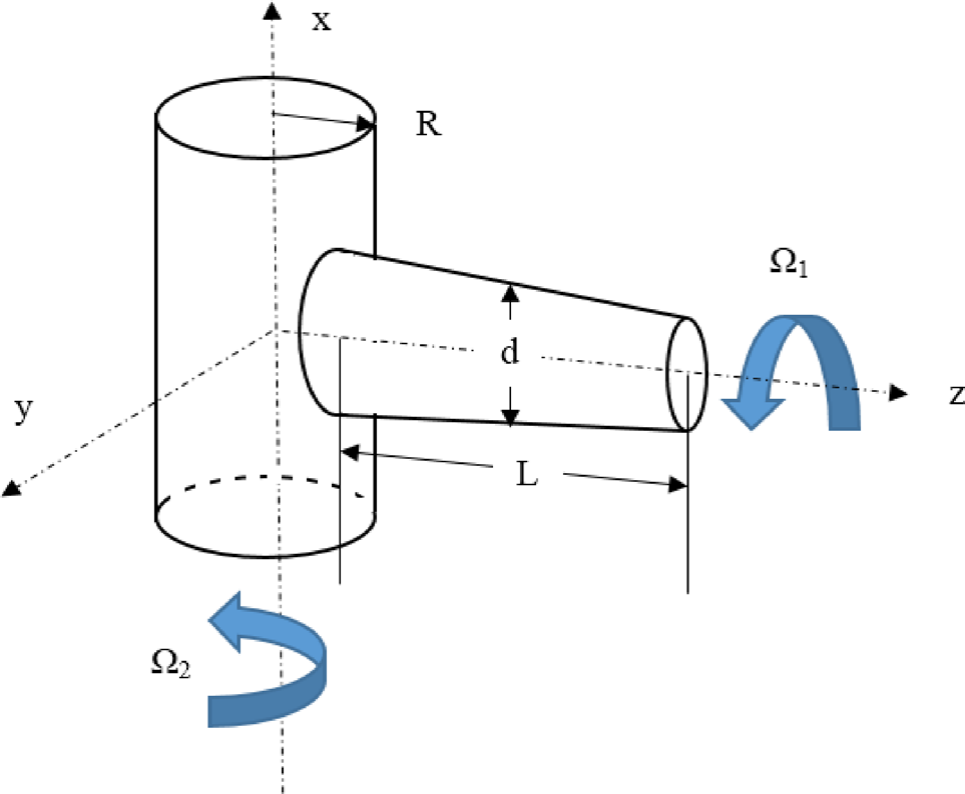
Geometrical characteristic of a nano-rotor.

As illustrated in Fig. 1, the system under consideration was a cantilevered nano-rotor with a variable diameter. The rotor extends along the longitudinal axis z with total length L, and its diameter d(z) decreases gradually along the span. The rotor was clamped at one end to a supporting shaft of radius R and remains free at the other end. The system underwent two simultaneous rotational motions. First, the nano-rotor rotated about its longitudinal axis (z-axis) with a constant angular velocity Ω_1_. Second, it rotated about the transverse axis (y-axis) with a constant angular velocity Ω_2_, representing a turbine-like motion. These combined rotational effects induced transverse displacements u_x_ and u_y_ in x- and y-directions, respectively, along with cross-sectional rotations ϕ_x_ and ϕ_y_. As a result, coupled bending and shear deformations were generated in x − z and y − z planes, which governed the dynamic behavior of the nano-rotor.

To clarify the rotational kinematics, the angular V vector of the nano-rotor was defined as the superposition of two perpendicular rotational components. The rotor spins about its longitudinal axis with angular V Ω_2_, while the supporting axis induces rotation about the transverse y-axis with angular V Ω_1_. Therefore, the angular V vector can be expressed as:

1 $$\Omega ={\Omega _1}{e_y}+{\Omega _2}{e_z}$$

where ey and ez are the unit vectors along the transverse and longitudinal axes, respectively. In this formulation, Ω_1_ contributes to the transverse rotation of the rotor about the supporting axis, whereas Ω_2_ represents the spin motion about the rotor axis. The inertial terms in the governing equations were obtained by considering the translational acceleration, rotary inertia, centrifugal stiffening, and gyroscopic coupling associated with this combined angular V field. The gyroscopic terms arose from the coupling between the spin motion and transverse bending rotations, while the transverse/support rotation generated the centrifugal axial force and varies along the rotor length. The vibrational characteristics and stability of the nano-rotor were substantially influenced by the gyroscopic effects, centrifugal stiffening, and NE introduced by the combined rotational motions. The mass distribution and stiffness were altered by variations in diameter and length, which, in turn, affected the critical Vs and the frequency divergence between forward and backward vibrational modes. Eringen’s NE theory and Timoshenko beam theory were employed to derive governing equations that faithfully represented these effects, accounting for both classical and nanoscale influences. DQM was employed to numerically solve these equations after they were transformed into a non-dimensional form. According to Timoshenko beam theory, and longitudinal deformation was small in the neutral axis, the displacement field in the following rotor will be as^26^:

2 $${{u_{zt}}\left( {x,z,t} \right)= - x{\varphi _y}\left( {z,t} \right)} \\ {{u_{xt}}\left( {x,z,t} \right)={u_x}\left( {z,t} \right)}$$

The displacement field of the nano-rotor is expressed using Timoshenko beam theory. As indicated in Eq. 2, the axial displacement component u_z_ (x, z,t) is given by −xϕ_y_ (z, t), where x denotes the distance from the neutral axis, and ϕ_y_ represents the rotation of the cross-section about the y-axis. The transverse displacement is defined as u_x_(x, z,t) = u_x_(z, t), indicating that it is independent of the thickness coordinate and varies only along the axial direction. Due to these displacement assumptions, non-zero strain components can be derived utilizing the strain–displacement relations of linear elasticity.

3 $$\begin{gathered} {\varepsilon _{zz}}= - x\frac{{\partial {\varphi _y}}}{{\partial z}} \hfill \\ {\gamma _{xz}}= - {\varphi _y}+\frac{{\partial {u_x}}}{{\partial z}} \hfill \\ \end{gathered}$$

In the above Eq. 3, the first term indicates the normal strain in the axial direction (ε_zz_), which depends on the distance from the neutral axis (x, the coordinate across the thickness measured from the neutral axis) and the beam curvature (∂ϕ_y_/∂_z_). Here, x is the position along the thickness of the beam, ϕ_y_(z, t) is the rotation of the cross-section about the y-axis, and ∂ϕy/∂z denotes the variation of this rotation along the axial coordinate z (the longitudinal axis of the nano-rotor). The second term expresses the shear strain in the x − z plane (γ_xz_) due to cross-sectional rotation and axial deformation. It consists of ϕy, representing the rotation of the beam’s cross-section, and ∂u_x_/∂z, where u_x_(z, t) is the transverse displacement in the x-direction and ∂ux/∂z indicates its change along with beam length z and represents the slope of transverse displacement, rather than an axial strain component. These equations together describe the strain behavior in a beam, incorporating both bending and shear effects, as captured by Timoshenko beam theory. Considering these two strain components, the corresponding non-zero stress values can be determined using Hooke’s law. The relationships involved the material properties: elastic modulus (E), shear modulus (G), and shear correction factor (k), which are related through Poisson’s ratio (ν). Using Hooke’s law, the resulting stress expressions based on Eq. 4 can be written as:

4 $$\begin{gathered} {\sigma _{zz}}=E{\varepsilon _{zz}} \hfill \\ {\sigma _{xz}}=kG{\gamma _{xz}} \hfill \\ \end{gathered}$$

Now, using Eqs. 3 and 4 will be written as:

5 $$\begin{gathered} {\sigma _{zz}}= - Ex\frac{{\partial {\varphi _y}}}{{\partial z}} \hfill \\ {\sigma _{xz}}=kG\left( { - {\varphi _y}+\frac{{\partial {u_x}}}{{\partial z}}} \right) \hfill \\ \end{gathered}$$

Bending moment (BM) and shear force in rotor sections are defined as:

6 $$\begin{gathered} {M_{yy}}= - EI\frac{{\partial {\varphi _y}}}{{\partial z}} \hfill \\ {Q_{xz}}=kGA\left( {\frac{{\partial {u_x}}}{{\partial z}} - {\varphi _y}} \right) \hfill \\ \end{gathered}$$

BM is denoted by the term M_yy_ = − EI(∂ϕ_y_/∂_z_). Here, E is the Young’s modulus and I = I(z) is the second moment of area of cross-section about the y-axis, which may vary along the axial coordinate z. The term ∂ϕ_y_/∂z represents the curvature of the beam, where ϕ_y_(z, t) is the rotation of the cross-section about the y-axis. The shear force is defined by Q_xz_=kGA(∂ux/∂z − ϕ_y_), where G is the shear modulus and A = A(z) is the cross-sectional area of the rotor. The coefficient k is the shear correction factor accounting for non-uniform shear stress distribution across the section. The term ∂u_x_/∂z denotes the variation of transverse displacement ux(z, t) along the axial direction, while ϕ_y_ represents the rotation of the cross-section contributing to shear deformation^3^.

7 $$\begin{gathered} {M_{yy}} - {\left( {{e_0}a} \right)^2}\frac{{{\partial ^2}{M_{yy}}}}{{\partial {z^2}}}= - EI\frac{{\partial {\varphi _y}}}{{\partial z}} \hfill \\ {Q_{xz}} - {\left( {{e_0}a} \right)^2}\frac{{{\partial ^2}{Q_{xz}}}}{{\partial {z^2}}}=kGA\left( {\frac{{\partial {u_x}}}{{\partial z}} - {\varphi _y}} \right) \hfill \\ \end{gathered}$$

**Fig. 2:**
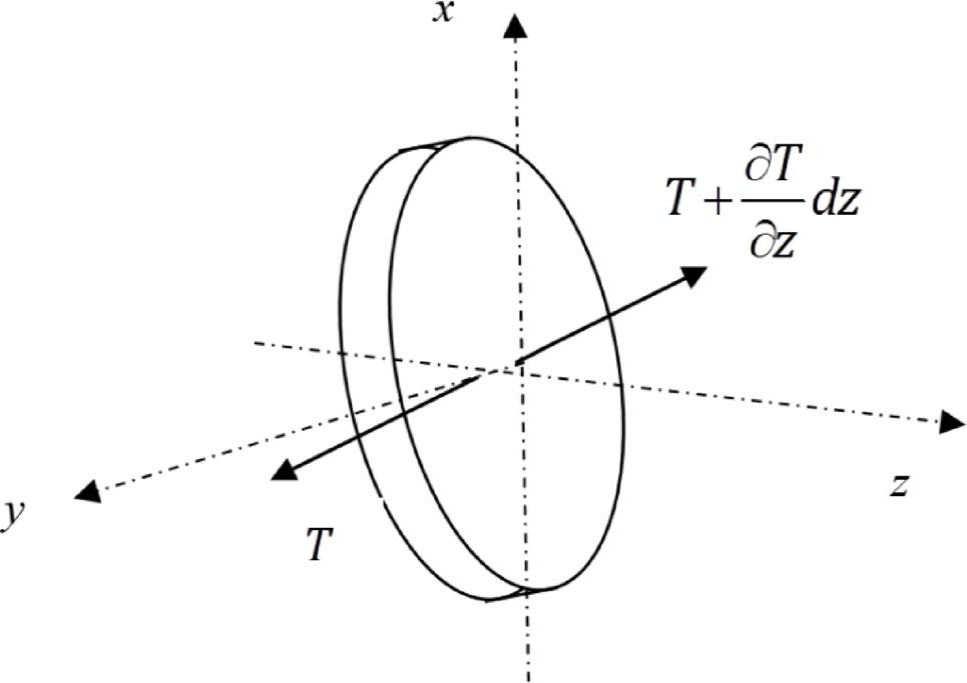
Axial tension force in the sample element of the rotor.

To incorporate small-scale impacts into the formulation, the differential form of Eringen’s NE theory was used. Accordingly, the non-local constitutive relation is expressed as:

8 $$(1 - {\mu ^2}{\nabla ^2}){\sigma _{ij}}={C_{ijkl}}{\varepsilon _{kl}}$$

where σ_ij_ and ε_kl_ denote the stress and strain tensors, respectively, C_ijkl_ is the elastic stiffness tensor, and µ = Le_0_a is the non-local parameter representing the small-scale impact. The operator ∇^2^ is the Laplacian, which introduces the effect of long-range interatomic interactions into the constitutive behavior. In the present beam formulation, this relation modifies the stress resultants by incorporating higher-order spatial derivatives, resulting in a non-local form of the governing equations. The effect of this rotation is an axial force which is variable with distance (*T*), shown in Fig. 2.

The amount of this force is calculated by Eq. 9^27^:

9 $$T\left( z \right)=\int\limits_{z}^{L} {\rho A\left( z \right)\Omega _{2}^{2}\left( {R+z} \right)dz}$$

In above equation *ρ*is the mass density of the rotor. According to Eq. 9, as the density, axis radius, and angular V increased, the amount of axial force rose, and this amount always decreased from a maximum amount at the clamped end (*z=0*) to reach zero at the other end (*z=L*). Considering this axial tension force, the definitions for BM elements and shear force were modified for a rotor^28^:

10 $$\begin{gathered} {M_x}=E{I_x}\frac{{\partial {\varphi _x}}}{{\partial z}} \hfill \\ {M_y}=E{I_y}\frac{{\partial {\varphi _y}}}{{\partial z}} \hfill \\ {F_x}=kGA\left( {\frac{{\partial {u_x}}}{{\partial z}} - {\varphi _y}} \right)+T\frac{{\partial {u_x}}}{{\partial z}} \hfill \\ {F_y}=kGA\left( {\frac{{\partial {u_y}}}{{\partial z}}+{\varphi _x}} \right)+T\frac{{\partial {u_y}}}{{\partial z}} \hfill \\ \end{gathered}$$

Considering Eringen’s theory, the components of BM and shear force will be stated as follows for a nano-rotor:

11 $$\begin{gathered} {M_x} - {\left( {{e_0}a} \right)^2}\frac{{{\partial ^2}{M_x}}}{{\partial {z^2}}}=E{I_x}\frac{{\partial {\varphi _x}}}{{\partial z}} \hfill \\ {M_y} - {\left( {{e_0}a} \right)^2}\frac{{{\partial ^2}{M_y}}}{{\partial {z^2}}}=E{I_y}\frac{{\partial {\varphi _y}}}{{\partial z}} \hfill \\ {F_x} - {\left( {{e_0}a} \right)^2}\frac{{{\partial ^2}{F_x}}}{{\partial {z^2}}}=kGA\left( {\frac{{\partial {u_x}}}{{\partial z}} - {\varphi _y}} \right)+T\frac{{\partial {u_x}}}{{\partial z}} \hfill \\ {F_y} - {\left( {{e_0}a} \right)^2}\frac{{{\partial ^2}{F_y}}}{{\partial {z^2}}}=kGA\left( {\frac{{\partial {u_y}}}{{\partial z}}+{\varphi _x}} \right)+T\frac{{\partial {u_y}}}{{\partial z}} \hfill \\ \end{gathered}$$

Using Newton’s second law and neglecting the weak terms (terms containing$d{z^2}$), the governing equations will be as:

12 $$\begin{gathered} \frac{{\partial {F_x}}}{{\partial z}}=\rho A\frac{{{\partial ^2}{u_x}}}{{\partial {t^2}}} \hfill \\ \frac{{\partial {F_y}}}{{\partial z}}=\rho A\frac{{{\partial ^2}{u_y}}}{{\partial {t^2}}} \hfill \\ \frac{{\partial {M_x}}}{{\partial z}} - {F_y}=\rho {I_x}\frac{{{\partial ^2}{\varphi _x}}}{{\partial {t^2}}}+\rho {I_p}{\Omega _1}\frac{{\partial {\varphi _y}}}{{\partial t}} \hfill \\ \frac{{\partial {M_y}}}{{\partial z}}+{F_x}=\rho {I_y}\frac{{{\partial ^2}{\varphi _y}}}{{\partial {t^2}}} - \rho {I_p}{\Omega _1}\frac{{\partial {\varphi _x}}}{{\partial t}} \hfill \\ \end{gathered}$$

In the above equation ${I_p}$is the rotor’s polar moment of inertia. It should be noted that in the last two Eqs. 11,12, the last term showed the gyroscopic effect. By differentiating Eq. 12 and putting the first two equations into the second two equations, it is concluded:

13 $$\begin{gathered} \frac{{{\partial ^2}{F_x}}}{{\partial {z^2}}}=\rho A\frac{{{\partial ^3}{u_x}}}{{\partial z\partial {t^2}}} \hfill \\ \frac{{{\partial ^2}{F_y}}}{{\partial {z^2}}}=\rho A\frac{{{\partial ^3}{u_y}}}{{\partial z\partial {t^2}}} \hfill \\ \frac{{{\partial ^2}{M_x}}}{{\partial {z^2}}}=\rho A\frac{{{\partial ^2}{u_y}}}{{\partial {t^2}}}+\rho {I_x}\frac{{{\partial ^3}{\varphi _x}}}{{\partial z\partial {t^2}}}+\rho {I_p}{\Omega _1}\frac{{{\partial ^2}{\varphi _y}}}{{\partial z\partial t}} \hfill \\ \frac{{{\partial ^2}{M_y}}}{{\partial {z^2}}}= - \rho A\frac{{{\partial ^2}{u_x}}}{{\partial {t^2}}}+\rho {I_y}\frac{{{\partial ^3}{\varphi _y}}}{{\partial z\partial {t^2}}} - \rho {I_p}{\Omega _1}\frac{{{\partial ^2}{\varphi _x}}}{{\partial z\partial t}} \hfill \\ \end{gathered}$$

Substituting Eq. (13) into Eq. (11), the components of BM and shear force will be as follows:

14 $$\begin{gathered} {M_x}=E{I_x}\frac{{\partial {\varphi _x}}}{{\partial z}}+{\left( {{e_0}a} \right)^2}\left( {\rho A\frac{{{\partial ^2}{u_y}}}{{\partial {t^2}}}+\rho {I_x}\frac{{{\partial ^3}{\varphi _x}}}{{\partial z\partial {t^2}}}+\rho {I_p}{\Omega _1}\frac{{{\partial ^2}{\varphi _y}}}{{\partial z\partial t}}} \right) \hfill \\ {M_y}=E{I_y}\frac{{\partial {\varphi _y}}}{{\partial z}}+{\left( {{e_0}a} \right)^2}\left( { - \rho A\frac{{{\partial ^2}{u_x}}}{{\partial {t^2}}}+\rho {I_y}\frac{{{\partial ^3}{\varphi _y}}}{{\partial z\partial {t^2}}} - \rho {I_p}{\Omega _1}\frac{{{\partial ^2}{\varphi _x}}}{{\partial z\partial t}}} \right) \hfill \\ {F_x}=kGA\left( {\frac{{\partial {u_x}}}{{\partial z}} - {\varphi _y}} \right)+T\frac{{\partial {u_x}}}{{\partial z}}+{\left( {{e_0}a} \right)^2}\rho A\frac{{{\partial ^3}{u_x}}}{{\partial z\partial {t^2}}} \hfill \\ {F_y}=kGA\left( {\frac{{\partial {u_y}}}{{\partial z}}+{\varphi _x}} \right)+T\frac{{\partial {u_y}}}{{\partial z}}+{\left( {{e_0}a} \right)^2}\rho A\frac{{{\partial ^3}{u_y}}}{{\partial z\partial {t^2}}} \hfill \\ \end{gathered}$$

For a circular cross-section, the following equations between bending and torsion moments are available:

15 $${I_p}=2{I_x}=2{I_y}=2I$$

The spatial derivative of this term explicitly appears in the governing equations and can be rigorously evaluated based on Eq. 10.

16 $$\frac{{\partial T}}{{\partial z}}= - \rho \,\Omega _{2}^{2}A\left( z \right)\left( {R+z} \right)$$

Now, putting Eqs. (10) and (16) into governing Eqs. (14), the governing equations will be rewritten as:

17 $$\begin{gathered} \left[ {kGA+\int\limits_{z}^{L} {\rho A\left( z \right)\Omega _{2}^{2}\left( {R+z} \right)dz} } \right]\frac{{{\partial ^2}{u_x}}}{{\partial {z^2}}}+kG\frac{{\partial A}}{{\partial z}}\frac{{\partial {u_x}}}{{\partial z}} - \rho \,\Omega _{2}^{2}A\left( z \right)\left( {R+z} \right)\frac{{\partial {u_x}}}{{\partial z}} \hfill \\ - kGA\frac{{\partial {\varphi _y}}}{{\partial z}} - kG\frac{{\partial A}}{{\partial z}}{\varphi _y} - \rho A\frac{{{\partial ^2}}}{{\partial {t^2}}}\left[ {{u_x} - {{\left( {{e_0}a} \right)}^2}\frac{{{\partial ^2}{u_x}}}{{\partial {z^2}}}} \right]=0 \hfill \\ \left[ {kGA+\int\limits_{z}^{L} {\rho A\left( z \right)\Omega _{2}^{2}\left( {R+z} \right)dz} } \right]\frac{{{\partial ^2}{u_y}}}{{\partial {z^2}}}+kG\frac{{\partial A}}{{\partial z}}\frac{{\partial {u_y}}}{{\partial z}} - \rho \,\Omega _{2}^{2}A\left( z \right)\left( {R+z} \right)\frac{{\partial {u_y}}}{{\partial z}} \hfill \\ +kGA\frac{{\partial {\varphi _x}}}{{\partial z}}+kG\frac{{\partial A}}{{\partial z}}{\varphi _x} - \rho A\frac{{{\partial ^2}}}{{\partial {t^2}}}\left[ {{u_y} - {{\left( {{e_0}a} \right)}^2}\frac{{{\partial ^2}{u_y}}}{{\partial {z^2}}}} \right]=0 \hfill \\ - \left[ {kGA+\int\limits_{z}^{L} {\rho A\left( z \right)\Omega _{2}^{2}\left( {R+z} \right)dz} } \right]\frac{{\partial {u_y}}}{{\partial z}}+EI\frac{{{\partial ^2}{\varphi _x}}}{{\partial {z^2}}}+E\frac{{\partial I}}{{\partial z}}\frac{{\partial {\varphi _x}}}{{\partial z}} - kGA{\varphi _x} \hfill \\ - 2\rho I{\Omega _1}\frac{\partial }{{\partial t}}\left[ {{\varphi _y} - {{\left( {{e_0}a} \right)}^2}\frac{{{\partial ^2}{\varphi _y}}}{{\partial {z^2}}}} \right] - \rho I\frac{{{\partial ^2}}}{{\partial {t^2}}}\left[ {{\varphi _x} - {{\left( {{e_0}a} \right)}^2}\frac{{{\partial ^2}{\varphi _x}}}{{\partial {z^2}}}} \right]=0 \hfill \\ \left[ {kGA+\int\limits_{z}^{L} {\rho A\left( z \right)\Omega _{2}^{2}\left( {R+z} \right)dz} } \right]\frac{{\partial {u_x}}}{{\partial z}}+EI\frac{{{\partial ^2}{\varphi _y}}}{{\partial {z^2}}}+E\frac{{\partial I}}{{\partial z}}\frac{{\partial {\varphi _y}}}{{\partial z}} - kGA{\varphi _y} \hfill \\ +2\rho I{\Omega _1}\frac{\partial }{{\partial t}}\left[ {{\varphi _x} - {{\left( {{e_0}a} \right)}^2}\frac{{{\partial ^2}{\varphi _x}}}{{\partial {z^2}}}} \right] - \rho I\frac{{{\partial ^2}}}{{\partial {t^2}}}\left[ {{\varphi _y} - {{\left( {{e_0}a} \right)}^2}\frac{{{\partial ^2}{\varphi _y}}}{{\partial {z^2}}}} \right]=0 \hfill \\ \end{gathered}$$

### Boundary conditions

The boundary conditions for the illustrated nano-rotor in Fig. 1 will be clamped. In*z=0*, due to being clamped, displacement and rotation components around *x* the *y* axes were zero.

18 $$\begin{array}{*{20}{c}} {{u_x}\left( 0 \right)=0}&{{u_y}\left( 0 \right)=0}&{{\varphi _x}\left( 0 \right)=0}&{{\varphi _y}\left( 0 \right)=0} \end{array}$$

In z = L, due to being free at this end, BM and shear force components around both *x* and *y* axes were zero.

19 $$\begin{array}{*{20}{c}} {{M_x}\left( L \right)=0}&{{M_y}\left( L \right)=0}&{{F_x}\left( L \right)=0}&{{F_y}\left( L \right)=0} \end{array}$$

Equation 10 demonstrates that the tension force at the end of the nano-rotor was zero. Consequently, boundary conditions could be stated in a simpler form:

20 $$\begin{gathered} EI\frac{{\partial {\varphi _x}}}{{\partial z}}+{\left( {{e_0}a} \right)^2}\left( {\rho A\frac{{{\partial ^2}{u_y}}}{{\partial {t^2}}}+\rho I\frac{{{\partial ^3}{\varphi _x}}}{{\partial z\partial {t^2}}}+2\rho I{\Omega _1}\frac{{{\partial ^2}{\varphi _y}}}{{\partial z\partial t}}} \right)=0 \hfill \\ EI\frac{{\partial {\varphi _y}}}{{\partial z}}+{\left( {{e_0}a} \right)^2}\left( { - \rho A\frac{{{\partial ^2}{u_x}}}{{\partial {t^2}}}+\rho I\frac{{{\partial ^3}{\varphi _y}}}{{\partial z\partial {t^2}}} - 2\rho I{\Omega _1}\frac{{{\partial ^2}{\varphi _x}}}{{\partial z\partial t}}} \right)=0 \hfill \\ kG\left( {\frac{{\partial {u_x}}}{{\partial z}} - {\varphi _y}} \right)+{\left( {{e_0}a} \right)^2}\rho \frac{{{\partial ^3}{u_x}}}{{\partial z\partial {t^2}}}=0 \hfill \\ kG\left( {\frac{{\partial {u_y}}}{{\partial z}}+{\varphi _x}} \right)+{\left( {{e_0}a} \right)^2}\rho \frac{{{\partial ^3}{u_y}}}{{\partial z\partial {t^2}}}=0 \hfill \\ \end{gathered}$$

### Dimensionless equation

To generalize the results, it would be better to present the equations and boundary conditions in non-dimensional form. For this purpose, assume a longitudinal non-dimensional variable defined as below:

21 $$0 \leqslant \zeta =\frac{z}{L} \leqslant 1$$

Now, applying the separation of variables method as below^29^:

22 $$\begin{array}{*{20}{c}} \begin{gathered} {u_x}\left( {\zeta ,t} \right)=L{v_x}\left( \zeta \right){e^{\omega \,t}} \hfill \\ {u_y}\left( {\zeta ,t} \right)=L{v_y}\left( \zeta \right){e^{\omega \,t}} \hfill \\ \end{gathered} \\ \begin{gathered} {\varphi _x}\left( {\zeta ,t} \right)={\psi _x}\left( \zeta \right){e^{\omega \,t}} \hfill \\ {\varphi _y}\left( {\zeta ,t} \right)={\psi _y}\left( \zeta \right){e^{\omega \,t}} \hfill \\ \end{gathered} \end{array}$$

The natural frequencies of the nano-rotor were identified from the associated eigenvalue problem. The corresponding non-dimensional forms of diameter, cross-sectional area, and BM are defined as follows:

23 $$\begin{gathered} d\left( \zeta \right)={d_0}{d^*}\left( \zeta \right) \hfill \\ A\left( \zeta \right)={A_0}{A^*}\left( \zeta \right) \hfill \\ I\left( \zeta \right)={I_0}{I^*}\left( \zeta \right) \hfill \\ \end{gathered}$$

The subscript 0 illustrates the corresponding values at the beginning and end of the nano-rotor (*z=0*). Now, the governing equations could be stated in a non-dimensional form as below:

24 $$\begin{gathered} \left[ {\frac{\alpha }{{{r^2}}}{A^*}\left( \zeta \right)+\gamma _{2}^{2}\int\limits_{\zeta }^{1} {{A^*}\left( \zeta \right)\left( {\delta +\zeta } \right)d\zeta } } \right]\frac{{{\partial ^2}{v_x}}}{{\partial {\zeta ^2}}}+\frac{\alpha }{{{r^2}}}\frac{{\partial {A^*}\left( \zeta \right)}}{{\partial \zeta }}\frac{{\partial {v_x}}}{{\partial \zeta }} \hfill \\ - \gamma _{2}^{2}{A^*}\left( \zeta \right)\left( {\delta +\zeta } \right)\frac{{\partial {v_x}}}{{\partial \zeta }} - \frac{\alpha }{{{r^2}}}{A^*}\left( \zeta \right)\frac{{\partial {\psi _y}}}{{\partial \zeta }} \hfill \\ - \frac{\alpha }{{{r^2}}}\frac{{\partial {A^*}\left( \zeta \right)}}{{\partial \zeta }}{\psi _y} - {\lambda ^2}{A^*}\left( \zeta \right)\left( {{v_x} - {\mu ^2}\frac{{{\partial ^2}{v_x}}}{{\partial {\zeta ^2}}}} \right)=0 \hfill \\ \left[ {\frac{\alpha }{{{r^2}}}{A^*}\left( \zeta \right)+\gamma _{2}^{2}\int\limits_{\zeta }^{1} {{A^*}\left( \zeta \right)\left( {\delta +\zeta } \right)d\zeta } } \right]\frac{{{\partial ^2}{v_y}}}{{\partial {\zeta ^2}}}+\frac{\alpha }{{{r^2}}}\frac{{\partial {A^*}\left( \zeta \right)}}{{\partial \zeta }}\frac{{\partial {v_y}}}{{\partial \zeta }} \hfill \\ - \gamma _{2}^{2}{A^*}\left( \zeta \right)\left( {\delta +\zeta } \right)\frac{{\partial {v_y}}}{{\partial \zeta }}+\frac{\alpha }{{{r^2}}}{A^*}\left( \zeta \right)\frac{{\partial {\psi _x}}}{{\partial \zeta }} \hfill \\ +\frac{\alpha }{{{r^2}}}\frac{{\partial {A^*}\left( \zeta \right)}}{{\partial \zeta }}{\psi _x} - {\lambda ^2}{A^*}\left( \zeta \right)\left( {{v_y} - {\mu ^2}\frac{{{\partial ^2}{v_y}}}{{\partial {\zeta ^2}}}} \right)=0 \hfill \\ - \left[ {\alpha {A^*}\left( \zeta \right)+{r^2}\gamma _{2}^{2}\int\limits_{z}^{L} {{A^*}\left( \zeta \right)\left( {\delta +\zeta } \right)d\zeta } } \right]\frac{{\partial {v_y}}}{{\partial \zeta }}+{r^2}{I^*}\left( \zeta \right)\frac{{{\partial ^2}{\psi _x}}}{{\partial {\zeta ^2}}}+{r^2}\frac{{\partial {I^*}\left( \zeta \right)}}{{\partial \zeta }}\frac{{\partial {\psi _x}}}{{\partial \zeta }} \hfill \\ - \alpha {A^*}\left( \zeta \right){\psi _x} - 2{r^4}{\gamma _1}\lambda {I^*}\left( \zeta \right)\left( {{\psi _y} - {\mu ^2}\frac{{{\partial ^2}{\psi _y}}}{{\partial {\zeta ^2}}}} \right) - {\lambda ^2}{r^4}{I^*}\left( \zeta \right)\left( {{\psi _x} - {\mu ^2}\frac{{{\partial ^2}{\psi _x}}}{{\partial {\zeta ^2}}}} \right)=0 \hfill \\ \left[ {\alpha {A^*}\left( \zeta \right)+{r^2}\gamma _{2}^{2}\int\limits_{z}^{L} {{A^*}\left( \zeta \right)\left( {\delta +\zeta } \right)d\zeta } } \right]\frac{{\partial {v_x}}}{{\partial \zeta }}+{r^2}{I^*}\left( \zeta \right)\frac{{{\partial ^2}{\psi _y}}}{{\partial {\zeta ^2}}}+{r^2}\frac{{\partial {I^*}\left( \zeta \right)}}{{\partial \zeta }}\frac{{\partial {\psi _y}}}{{\partial \zeta }} \hfill \\ - \alpha {A^*}\left( \zeta \right){\psi _y}+2{r^4}{\gamma _1}\lambda {I^*}\left( \zeta \right)\left( {{\psi _x} - {\mu ^2}\frac{{{\partial ^2}{\psi _x}}}{{\partial {\zeta ^2}}}} \right) - {\lambda ^2}{r^4}{I^*}\left( \zeta \right)\left( {{\psi _y} - {\mu ^2}\frac{{{\partial ^2}{\psi _y}}}{{\partial {\zeta ^2}}}} \right)=0 \hfill \\ \end{gathered}$$

Where the following non-dimensional variables are defined:

25 $$\begin{array}{*{20}{c}} {\begin{array}{*{20}{c}} {\alpha =\frac{{kG}}{E}}&{{r^2}=\frac{{{I_0}}}{{{A_0}{L^2}}}}&{\delta =\frac{R}{L}}&{\mu =\frac{{{e_0}a}}{L}} \end{array}} \\ {\begin{array}{*{20}{c}} {\gamma _{1}^{2}=\frac{{\rho {A_0}{L^4}\Omega _{1}^{2}}}{{E{I_0}}}}&{\gamma _{2}^{2}=\frac{{\rho {A_0}\,{L^4}\Omega _{2}^{2}}}{{E{I_0}}}}&{{\lambda ^2}=\frac{{\rho {A_0}{L^4}{\omega ^2}}}{{E{I_0}}}} \end{array}} \end{array}$$

Among the presented non-dimensional variables in Eq. 25, ${\gamma _1}$and ${\gamma _2}$ are non-dimensional rotational Vs around *x* and *z* axes, and *λ* is a non-dimensional form of frequency and nano-rotor vibrations, which is the indefinite parameter in this problem. Using the equations below, two non-dimensional parameters *α* and *r* are stated:

26 $$\begin{gathered} \begin{array}{*{20}{c}} {G=\frac{E}{{2\left( {1+\upsilon } \right)}}}&{{A_0}=\frac{\pi }{4}d_{0}^{2}}&{{I_0}=\frac{\pi }{{64}}d_{0}^{4}} \end{array} \hfill \\ \begin{array}{*{20}{c}} {\alpha =\frac{{3\left( {1+\upsilon } \right)}}{{7+12\upsilon +4{\upsilon ^2}}}}&{r=\frac{{{d_0}}}{{4L}}} \end{array} \hfill \\ \end{gathered}$$

It could be stated that *r* is a non-dimensional slenderness ratio, which means that for longer and thinner rotors, this parameter is less, and the Euler-Bernoulli model, in which shear deformations and moment of inertia were neglected, would be better in this case.

27 $$\begin{array}{l} \begin{array}{*{20}{c}} {\zeta = 0:}&{\left\{ {\begin{array}{*{20}{c}} {{v_x} = 0}\\ {{v_y} = 0}\\ {{\psi _x} = 0}\\ {{\psi _y} = 0} \end{array}} \right.} \end{array}\\ \begin{array}{*{20}{c}} {\zeta = 1:}&{\left\{ {\begin{array}{*{20}{c}} {\frac{{\partial {\psi _x}}}{{\partial \zeta }} + 2{\mu ^2}{r^2}{\gamma _1}\lambda \frac{{\partial {\psi _y}}}{{\partial \zeta }} + {\mu ^2}{\lambda ^2}\left( {{\beta ^{ - 2}}{v_y} + {r^2}\frac{{\partial {\psi _x}}}{{\partial \zeta }}} \right) = 0}\\ {\frac{{\partial {\psi _y}}}{{\partial \zeta }} - 2{\mu ^2}{r^2}{\gamma _1}\lambda \frac{{\partial {\psi _x}}}{{\partial \zeta }} + {\mu ^2}{\lambda ^2}\left( { - {\beta ^{ - 2}}{v_x} + {r^2}\frac{{\partial {\psi _y}}}{{\partial \zeta }}} \right) = 0}\\ {\frac{\alpha }{{{r^2}}}\left( {\frac{{\partial {v_x}}}{{\partial \zeta }} - {\psi _y}} \right) + {\mu ^2}{\lambda ^2}\frac{{\partial {v_x}}}{{\partial \zeta }} = 0}\\ {\frac{\alpha }{{{r^2}}}\left( {\frac{{\partial {v_y}}}{{\partial \zeta }} + {\psi _x}} \right) + {\mu ^2}{\lambda ^2}\frac{{\partial {v_y}}}{{\partial \zeta }} = 0} \end{array}} \right.} \end{array} \end{array}$$

The parameter *β* is defined as the diameter ratio of the nano-rotor between axial positions *z=L*and *z=0*, i.e., it characterizes the variation of the rotor diameter along its length.

28 $$\beta =\frac{d_1}{d_0}={d^*}(1)$$

### DQM

The differential quadrature method (DQM) was a numerical technique for approximating derivatives by expressing them as weighted sums of function values at discrete points. In contrast to conventional finite difference methods, DQM achieved high accuracy with a relatively small number of grid points using weighting coefficients (WCs) derived from interpolation polynomials. This method was widely used in vibration analysis, fluid dynamics, and structural mechanics. The first-order derivative WCs in DQM are defined as follows:

29 $$A_{{ij}}^{{^{{(1)}}}}=\left\{ \begin{gathered} \frac{{\prod\limits_{\begin{subarray}{l} m=1 \\ m \ne i,j \end{subarray} }^{N} {({x_i} - {x_m})} }}{{\prod\limits_{\begin{subarray}{l} m=1 \\ m \ne j \end{subarray} }^{N} {({x_j} - {x_m})} }}\,\,\,,\,\,\,(i,j=1,2,3,...,N;\,i \ne j) \hfill \\ \sum\limits_{\begin{subarray}{l} m=1 \\ m \ne i \end{subarray} }^{N} {\frac{1}{{({x_i} - {x_m})}}\,\,,\,\,\,(i=j=1,2,3,...,N)} \hfill \\ \end{gathered} \right.$$

The WCs matrix for the first derivative is denoted by A_ij_(1) in the aforementioned equation. This enabled the function’s derivative to be expressed as a weighted sum of its values at specific grid points. The discrete grid points were represented by the variables x_i_ and x_j_. x_i_ was the location at which the derivative was evaluated, while x_j_ represented the other points within the domain. The total number of grid points, N determined the resolution and accuracy of the numerical approximation. The equation was composed of two cases: when ≠, the coefficient was calculated using a product formula derived from Lagrange interpolation polynomials, ensuring that all grid points except x_j_ contributed to the approximation. This formulation guaranteed a high degree of precision in derivative estimation. The equation contained a summation term when i = j, which ensured numerical consistency by appropriately defining the diagonal elements of the coefficient matrix^29^.To better understand Eq. 29, the matrix form of it could be used:

30 $$\left\{ {\frac{{df}}{{dx}}} \right\}=\left[ {{A^{\left( 1 \right)}}} \right]\left\{ f \right\}$$

Since the second derivative is gained through differentiation of the first derivative, it is concluded:

31 $$\left\{ {\frac{{{d^2}f}}{{d{x^2}}}} \right\}=\left[ {{A^{\left( 1 \right)}}} \right]\left\{ {\frac{{df}}{{dx}}} \right\}=\left[ {{A^{\left( 1 \right)}}} \right]\left[ {{A^{\left( 1 \right)}}} \right]\left\{ f \right\}=\left[ {{A^{\left( 2 \right)}}} \right]\left\{ f \right\}$$

And in general,

32 $$\begin{array}{*{20}{c}} {\left\{ {\frac{{{d^r}f}}{{d{x^r}}}} \right\}=\left[ {{A^{\left( r \right)}}} \right]\left\{ f \right\}}&{{A^{(r)}}={A^{(1)}}{A^{(r - 1)}}} \end{array}.$$

It should be noted that to make the symbolism simple in this article, weight coefficient matrices for two derivatives are named as follows:

33 $$\begin{array}{*{20}{c}} {\left[ A \right]=\left[ {{A^{\left( 1 \right)}}} \right]}&{\left[ B \right]=\left[ {{A^{\left( 2 \right)}}} \right]} \end{array}.$$

Consequently,

34 $$\begin{array}{*{20}{c}} {\left\{ {\frac{{df}}{{dx}}} \right\}=\left[ A \right]\left\{ f \right\}}&{\left\{ {\frac{{{d^2}f}}{{d{x^2}}}} \right\}=\left[ B \right]\left\{ f \right\}} \end{array}.$$

The accuracy increased as the number of points in the domain increased, and eventually, the required convergence was achieved with a specific number of points. In addition to the quantity of points, their distribution within the domain was significant. A cosine distribution known as the Chebyshev Gauss Lobatto grid was the most effective distribution that was identified thus far. The most critical characteristic of this distribution was that the density of points on the boundaries was greater than that of the points in the interior. Consequently, the function was approximated with more points in areas where abrupt changes were anticipated, thereby improving accuracy.

This approximation is calculated via the following equation:

35 $$\begin{array}{*{20}{c}} {{{\overline {x} }_i}=\frac{{{x_i}}}{L}=\frac{1}{2}\left\{ {1 - \cos \left[ {\frac{{\left( {i - 1} \right)\pi }}{{N - 1}}} \right]} \right\}}&{i=1,2,3,...,N} \end{array}$$

Where L is the length of the domain.

#### DQM solution

After discretizing the non-dimensional governing equations (Eq. 24) using the DQM weighting matrices (Eqs. 29–34), the resulting system of algebraic equations. is obtained. These equations are then assembled in matrix form as follows:

36 $$\begin{gathered} \left( {\frac{\alpha }{{{r^2}}}\left[ a \right]+\gamma _{2}^{2}\left[ f \right]} \right)\left[ B \right]\left\{ {{v_x}} \right\}+\frac{\alpha }{{{r^2}}}\left[ b \right]\left[ A \right]\left\{ {{v_x}} \right\} - \gamma _{2}^{2}\left[ e \right]\left[ A \right]\left\{ {{v_x}} \right\} \hfill \\ - \frac{\alpha }{{{r^2}}}\left[ a \right]\left[ A \right]\left\{ {{\psi _y}} \right\} - \frac{\alpha }{{{r^2}}}\left[ b \right]\left\{ {{\psi _y}} \right\} - {\lambda ^2}\left[ a \right]\left( {\left\{ {{v_x}} \right\} - {\mu ^2}\left[ B \right]\left\{ {{v_x}} \right\}} \right)=0 \hfill \\ \left( {\frac{\alpha }{{{r^2}}}\left[ a \right]+\gamma _{2}^{2}\left[ f \right]} \right)\left[ B \right]\left\{ {{v_y}} \right\}+\frac{\alpha }{{{r^2}}}\left[ b \right]\left[ A \right]\left\{ {{v_y}} \right\} - \gamma _{2}^{2}\left[ e \right]\left[ A \right]\left\{ {{v_y}} \right\} \hfill \\ +\frac{\alpha }{{{r^2}}}\left[ a \right]\left[ A \right]\left\{ {{\psi _x}} \right\}+\frac{\alpha }{{{r^2}}}\left[ b \right]\left\{ {{\psi _x}} \right\} - {\lambda ^2}\left[ a \right]\left( {\left\{ {{v_y}} \right\} - {\mu ^2}\left[ B \right]\left\{ {{v_y}} \right\}} \right)=0 \hfill \\ - \left( {\alpha \left[ a \right]+{r^2}\gamma _{2}^{2}\left[ f \right]} \right)\left[ A \right]\left\{ {{v_y}} \right\}+{r^2}\left[ c \right]\left[ B \right]\left\{ {{\psi _x}} \right\}+{r^2}\left[ d \right]\left[ A \right]\left\{ {{\psi _x}} \right\} - \alpha \left[ a \right]\left\{ {{\psi _x}} \right\} \hfill \\ - 2{r^4}{\gamma _1}\lambda \left[ c \right]\left( {\left\{ {{\psi _y}} \right\} - {\mu ^2}\left[ B \right]\left\{ {{\psi _y}} \right\}} \right) - {\lambda ^2}{r^4}\left[ c \right]\left( {\left\{ {{\psi _x}} \right\} - {\mu ^2}\left[ B \right]\left\{ {{\psi _x}} \right\}} \right)=0 \hfill \\ \left( {\alpha \left[ a \right]+{r^2}\gamma _{2}^{2}\left[ f \right]} \right)\left[ A \right]\left\{ {{v_x}} \right\}+{r^2}\left[ c \right]\left[ B \right]\left\{ {{\psi _y}} \right\}+{r^2}\left[ d \right]\left[ A \right]\left\{ {{\psi _y}} \right\} - \alpha \left[ a \right]\left\{ {{\psi _y}} \right\} \hfill \\ +2{r^4}{\gamma _1}\lambda \left[ c \right]\left( {\left\{ {{\psi _x}} \right\} - {\mu ^2}\left[ B \right]\left\{ {{\psi _x}} \right\}} \right) - {\lambda ^2}{r^4}\left[ c \right]\left( {\left\{ {{\psi _y}} \right\} - {\mu ^2}\left[ B \right]\left\{ {{\psi _y}} \right\}} \right)=0 \hfill \\ \end{gathered}$$

In which matrices $\left[ A \right]$and $\left[ B \right]$are square matrices with the order of *N* which are corresponding with the first and second derivatives in DQM, respectively and matrices $\left[ a \right]$to $\left[ f \right]$are diagonal matrices with the order of *N* which are defined as:

37 $${\left\{ d \right\}_{4N \times 1}}=\left\{ {\begin{array}{*{20}{c}} {{{\left\{ {{v_x}} \right\}}_{N \times 1}}} \\ {{{\left\{ {{v_y}} \right\}}_{N \times 1}}} \\ {{{\left\{ {{\psi _x}} \right\}}_{N \times 1}}} \\ {{{\left\{ {{\psi _y}} \right\}}_{N \times 1}}} \end{array}} \right\}$$

In Eq. 37, v_x_ and v_y_ are defined as *N*×1 vectors representing the non-dimensional transverse displacement shape functions in the x and y directions, respectively. Similarly, Ψ_x_ and Ψ_y_ are *N*×1 vectors corresponding to the rotational shape functions of the beam cross-section about x and y axes. These variables originated from the separation of variables applied to the displacement and rotation fields (Eq. 22) and therefore did not represent V components or fluid-related quantities. In the differential quadrature method, differentiation matrices play a central role in transforming the governing equations into a discrete form. The spatial derivatives of the system variables are approximated using WC matrices D1 and D2, corresponding to first- and second-order derivatives, respectively. Using these matrices to transform the non-dimensional governing equations, the continuous system was reduced to a set of coupled algebraic equations. The discretized system describes the dynamic behavior of the nano-rotor and can be expressed as a generalized eigenvalue problem, where the eigenvalues determine the natural frequencies, and the associated eigenvectors define the corresponding mode shapes.

38 $${\lambda ^2}\left[ M \right]\left\{ d \right\}+\lambda \left[ C \right]\left\{ d \right\}+\left[ K \right]\left\{ d \right\}=\left\{ 0 \right\}$$

In Eq. 38, [M], [C], and [K] denote the mass, damping, and stiffness matrices of the system, respectively. These matrices represented the inertial, dissipative, and restoring characteristics governing the system’s dynamic response. The eigenvalue parameter λ determines the natural frequencies, while the associated eigenvectors define the corresponding mode shapes. The displacement vector [d] includes the discretized non-dimensional transverse displacement functions (v_x_, v_y_) and the rotational functions (Ψ_x_, Ψ_y_).

39 $$\begin{gathered} \left[ K \right]=\left[ {\begin{array}{*{20}{c}} {{K_{11}}}&{\left[ 0 \right]}&{\left[ 0 \right]}&{{K_{14}}} \\ {\left[ 0 \right]}&{{K_{22}}}&{{K_{23}}}&{\left[ 0 \right]} \\ {\left[ 0 \right]}&{{K_{32}}}&{{K_{33}}}&{\left[ 0 \right]} \\ {{K_{41}}}&{\left[ 0 \right]}&{\left[ 0 \right]}&{{K_{44}}} \end{array}} \right] \hfill \\ \left[ C \right]=\left[ {\begin{array}{*{20}{c}} {\left[ 0 \right]}&{\left[ 0 \right]}&{\left[ 0 \right]}&{\left[ 0 \right]} \\ {\left[ 0 \right]}&{\left[ 0 \right]}&{\left[ 0 \right]}&{\left[ 0 \right]} \\ {\left[ 0 \right]}&{\left[ 0 \right]}&{\left[ 0 \right]}&{{C_{34}}} \\ {\left[ 0 \right]}&{\left[ 0 \right]}&{{C_{43}}}&{\left[ 0 \right]} \end{array}} \right] \hfill \\ \left[ M \right]=\left[ {\begin{array}{*{20}{c}} {{M_{11}}}&{\left[ 0 \right]}&{\left[ 0 \right]}&{\left[ 0 \right]} \\ {\left[ 0 \right]}&{{M_{11}}}&{\left[ 0 \right]}&{\left[ 0 \right]} \\ {\left[ 0 \right]}&{\left[ 0 \right]}&{{M_{33}}}&{\left[ 0 \right]} \\ {\left[ 0 \right]}&{\left[ 0 \right]}&{\left[ 0 \right]}&{{M_{44}}} \end{array}} \right] \hfill \\ \end{gathered}$$

Regarding Eq. 39, $\left[ 0 \right]$ shows a zero square matrix with the order of *N*, and the following matrices are defined as:

40 $$\begin{gathered} {K_{11}}={K_{22}}=\left( {\frac{\alpha }{{{r^2}}}\left[ a \right]+\gamma _{2}^{2}\left[ f \right]} \right)\left[ B \right]+\left( {\frac{\alpha }{{{r^2}}}\left[ b \right] - \gamma _{2}^{2}\left[ e \right]} \right)\left[ A \right] \hfill \\ {K_{33}}={K_{44}}={r^2}\left( {\left[ c \right]\left[ B \right]+\left[ d \right]\left[ A \right]} \right) - \alpha \left[ a \right] \hfill \\ {K_{14}}= - \frac{\alpha }{{{r^2}}}\left( {\left[ a \right]\left[ A \right]+\left[ b \right]} \right) \hfill \\ {C_{34}}= - 2{r^4}{\gamma _1}\left[ c \right]\left( {I - {\mu ^2}\left[ B \right]} \right) \hfill \\ {C_{43}}= - {C_{34}} \hfill \\ {M_{11}}={M_{22}}= - \left[ a \right]\left( {I - {\mu ^2}\left[ B \right]} \right) \hfill \\ {M_{33}}={M_{44}}= - {r^4}\left[ c \right]\left( {I - {\mu ^2}\left[ B \right]} \right) \hfill \\ \end{gathered}$$

The non-dimensional form of the boundary conditions was derived from Eq. (27) presented. At this point, it can be rewritten as follows using Eq. (34).

41 $${\lambda ^2}\left[ p \right]\left\{ d \right\}+\lambda \left[ q \right]\left\{ d \right\}+\left[ r \right]\left\{ d \right\}=\left\{ 0 \right\}$$

In which matrices$\left[ p \right]$, $\left[ q \right]$, and $\left[ r \right]$are in 8 × 4 N order and will be defined as:

42 $$\begin{gathered} \left[ p \right]={\mu ^2}\left[ {\begin{array}{*{20}{c}} {{{\left\{ 0 \right\}}_{1 \times N}}}&{{{\left\{ 0 \right\}}_{1 \times N}}}&{{{\left\{ 0 \right\}}_{1 \times N}}}&{{{\left\{ 0 \right\}}_{1 \times N}}} \\ {{{\left\{ 0 \right\}}_{1 \times N}}}&{{{\left\{ 0 \right\}}_{1 \times N}}}&{{{\left\{ 0 \right\}}_{1 \times N}}}&{{{\left\{ 0 \right\}}_{1 \times N}}} \\ {{{\left\{ 0 \right\}}_{1 \times N}}}&{{{\left\{ 0 \right\}}_{1 \times N}}}&{{{\left\{ 0 \right\}}_{1 \times N}}}&{{{\left\{ 0 \right\}}_{1 \times N}}} \\ {{{\left\{ 0 \right\}}_{1 \times N}}}&{{{\left\{ 0 \right\}}_{1 \times N}}}&{{{\left\{ 0 \right\}}_{1 \times N}}}&{{{\left\{ 0 \right\}}_{1 \times N}}} \\ {{{\left\{ 0 \right\}}_{1 \times N}}}&{{\beta ^{ - 2}}{I_{Nj}}}&{{r^2}{A_{Nj}}}&{{{\left\{ 0 \right\}}_{1 \times N}}} \\ { - {\beta ^{ - 2}}{I_{Nj}}}&{{{\left\{ 0 \right\}}_{1 \times N}}}&{{{\left\{ 0 \right\}}_{1 \times N}}}&{{r^2}{A_{Nj}}} \\ {{A_{Nj}}}&{{{\left\{ 0 \right\}}_{1 \times N}}}&{{{\left\{ 0 \right\}}_{1 \times N}}}&{{{\left\{ 0 \right\}}_{1 \times N}}} \\ {{{\left\{ 0 \right\}}_{1 \times N}}}&{{A_{Nj}}}&{{{\left\{ 0 \right\}}_{1 \times N}}}&{{{\left\{ 0 \right\}}_{1 \times N}}} \end{array}} \right] \hfill \\ \left[ q \right]=2{\mu ^2}{r^2}{\gamma _1}\left[ {\begin{array}{*{20}{c}} {{{\left\{ 0 \right\}}_{1 \times N}}}&{{{\left\{ 0 \right\}}_{1 \times N}}}&{{{\left\{ 0 \right\}}_{1 \times N}}}&{{{\left\{ 0 \right\}}_{1 \times N}}} \\ {{{\left\{ 0 \right\}}_{1 \times N}}}&{{{\left\{ 0 \right\}}_{1 \times N}}}&{{{\left\{ 0 \right\}}_{1 \times N}}}&{{{\left\{ 0 \right\}}_{1 \times N}}} \\ {{{\left\{ 0 \right\}}_{1 \times N}}}&{{{\left\{ 0 \right\}}_{1 \times N}}}&{{{\left\{ 0 \right\}}_{1 \times N}}}&{{{\left\{ 0 \right\}}_{1 \times N}}} \\ {{{\left\{ 0 \right\}}_{1 \times N}}}&{{{\left\{ 0 \right\}}_{1 \times N}}}&{{{\left\{ 0 \right\}}_{1 \times N}}}&{{{\left\{ 0 \right\}}_{1 \times N}}} \\ {{{\left\{ 0 \right\}}_{1 \times N}}}&{{{\left\{ 0 \right\}}_{1 \times N}}}&{{{\left\{ 0 \right\}}_{1 \times N}}}&{{A_{Nj}}} \\ {{{\left\{ 0 \right\}}_{1 \times N}}}&{{{\left\{ 0 \right\}}_{1 \times N}}}&{ - {A_{Nj}}}&{{{\left\{ 0 \right\}}_{1 \times N}}} \\ {{{\left\{ 0 \right\}}_{1 \times N}}}&{{{\left\{ 0 \right\}}_{1 \times N}}}&{{{\left\{ 0 \right\}}_{1 \times N}}}&{{{\left\{ 0 \right\}}_{1 \times N}}} \\ {{{\left\{ 0 \right\}}_{1 \times N}}}&{{{\left\{ 0 \right\}}_{1 \times N}}}&{{{\left\{ 0 \right\}}_{1 \times N}}}&{{{\left\{ 0 \right\}}_{1 \times N}}} \end{array}} \right] \hfill \\ \left[ r \right]=\left[ {\begin{array}{*{20}{c}} {{I_{1j}}}&{{{\left\{ 0 \right\}}_{1 \times N}}}&{{{\left\{ 0 \right\}}_{1 \times N}}}&{{{\left\{ 0 \right\}}_{1 \times N}}} \\ {{{\left\{ 0 \right\}}_{1 \times N}}}&{{I_{1j}}}&{{{\left\{ 0 \right\}}_{1 \times N}}}&{{{\left\{ 0 \right\}}_{1 \times N}}} \\ {{{\left\{ 0 \right\}}_{1 \times N}}}&{{{\left\{ 0 \right\}}_{1 \times N}}}&{{I_{1j}}}&{{{\left\{ 0 \right\}}_{1 \times N}}} \\ {{{\left\{ 0 \right\}}_{1 \times N}}}&{{{\left\{ 0 \right\}}_{1 \times N}}}&{{{\left\{ 0 \right\}}_{1 \times N}}}&{{I_{1j}}} \\ {{{\left\{ 0 \right\}}_{1 \times N}}}&{{{\left\{ 0 \right\}}_{1 \times N}}}&{{A_{Nj}}}&{{{\left\{ 0 \right\}}_{1 \times N}}} \\ {{{\left\{ 0 \right\}}_{1 \times N}}}&{{{\left\{ 0 \right\}}_{1 \times N}}}&{{{\left\{ 0 \right\}}_{1 \times N}}}&{{A_{Nj}}} \\ {\frac{\alpha }{{{r^2}}}{A_{Nj}}}&{{{\left\{ 0 \right\}}_{1 \times N}}}&{{{\left\{ 0 \right\}}_{1 \times N}}}&{ - \frac{\alpha }{{{r^2}}}{I_{Nj}}} \\ {{{\left\{ 0 \right\}}_{1 \times N}}}&{\frac{\alpha }{{{r^2}}}{A_{Nj}}}&{\frac{\alpha }{{{r^2}}}{I_{Nj}}}&{{{\left\{ 0 \right\}}_{1 \times N}}} \end{array}} \right] \hfill \\ \end{gathered}$$

In which [0] represented a zero vector of order 1×N and the subscripts 1j and Nj showed the first and last rows of each matrix, respectively. Since satisfying the governing equations and boundary conditions simultaneously led to more equations than unknown parameters and, in turn, created non-square matrices, it was crucial to neglect satisfying the governing equations at boundary points^30–32^. The boundary points are considered as follows:

43 $${\left\{ d \right\}_b}={\left\{ {\begin{array}{*{20}{c}} {{d_1}}&{{d_N}}&{{d_{N+1}}}&{{d_{2N}}}&{{d_{2N+1}}}&{{d_{3N}}}&{{d_{3N+1}}}&{{d_{4N}}} \end{array}} \right\}^T}$$

The other points were domain points, which are shown as ${\left\{ {\left. d \right\}} \right._d}$. Boundary points should be equal to boundary conditions so that the final matrix is square. Omitting the equations in boundary points, Eq. (36) will be stated as follows:

44 $${\lambda ^2}\left[ {\bar {M}} \right]\left\{ d \right\}+\lambda \left[ {\bar {C}} \right]\left\{ d \right\}+\left[ {\bar {K}} \right]\left\{ d \right\}=0$$

In this formulation, the overbar notation denoted the corresponding non-square matrices of appropriate dimensions. By incorporating the boundary conditions (Eq. 41) into this system, the governing equation can be expressed in the following form:

45 $${\lambda ^2}\left[ m \right]\left\{ d \right\}+\lambda \left[ c \right]\left\{ d \right\}+\left[ k \right]\left\{ d \right\}=0$$

Where

46 $$\begin{array}{*{20}{c}} {\left[ m \right]=\left[ {\begin{array}{*{20}{c}} {\bar {M}} \\ p \end{array}} \right]}&{\left[ c \right]=\left[ {\begin{array}{*{20}{c}} {\bar {C}} \\ q \end{array}} \right]}&{\left[ k \right]=\left[ {\begin{array}{*{20}{c}} {\bar {K}} \\ r \end{array}} \right]} \end{array}$$

Equation (45) is a non-standard eigenvalue problem that could turn into a standard one by defining a new displacement vector $\left\{ {\left. X \right\}} \right.$:

47 $$\begin{gathered} \left\{ X \right\}=\left\{ {\begin{array}{*{20}{c}} {\left\{ d \right\}} \\ {\lambda \left\{ d \right\}} \end{array}} \right\} \hfill \\ \left[ {{F_1}} \right]\left\{ X \right\}=\lambda \left[ {{F_2}} \right]\left\{ X \right\} \hfill \\ \end{gathered}$$

Where

48 $$\begin{array}{*{20}{c}} {\left[ {{F_1}} \right]=\left[ {\begin{array}{*{20}{c}} {\left[ 0 \right]}&I \\ {\left[ k \right]}&{\left[ c \right]} \end{array}} \right]}&{\left[ {{F_2}} \right]=\left[ {\begin{array}{*{20}{c}} I&{\left[ 0 \right]} \\ {\left[ 0 \right]}&{ - \left[ m \right]} \end{array}} \right]} \end{array}$$

In this equation, $\left[ 0 \right]$and [*I*] are zero and identity matrices of *4N* the same order. In the final Eq. (47), the acquired matrices are square with *8N* order. In solving this eigenvalue problem, the non-dimensional frequencies *λ*were gained. While the rotor was vibrating, two motions happened: the rotation of the axis with an angular V of ${\Omega _1}$ (non-dimensional${\gamma _1}$) and the bending of the axis (transverse vibration in two planes) with a frequency of *ω* (non-dimensional *λ*). If these two motions were in the same direction$\lambda \gamma \rangle 0$, it was called forward motion, and the corresponding frequency was called forward frequency. Otherwise (if $\lambda \gamma \langle 0$), the motion was backward, and the corresponding frequency will naturally be called the backward frequency.

## Validation

**Fig. 3:**
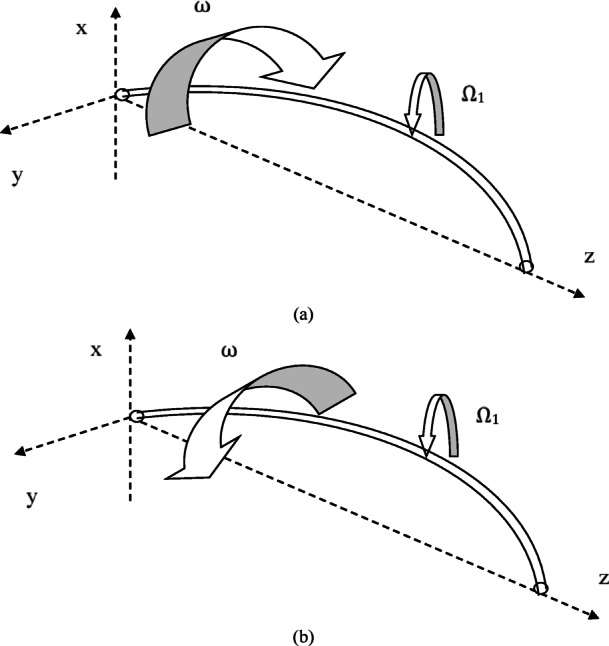
**(a)** Forward frequencies, and **(b)** backward frequencies.

The convergence and validity of results should be verified, as the method being presented was numerical. Subsequently, the critical Vs and the forward and backward frequencies were examined with respect to various problem variables. The forward and reverse frequencies are illustrated in Fig. 3.

In all of the instances, the Poisson’s ratio was assumed to be 0.3. Before delving into the findings of any numerical study, it was essential to ensure their convergence and validity. This section addresses these two difficulties. Consider a nano-rotor with the following properties to test the convergence of the numerical approach described here and determine the number of points necessary to solve the problem:

49 $$\begin{array}{*{20}{c}} {{d^*}\left( \zeta \right)=\exp \left( { - 0.1\zeta } \right)}&{,\delta =0.25}&{,{\gamma _1}=5}&{,{\gamma _2}=3}&{,r=0.03}&{,\mu =0.1} \end{array}$$

The first four values of forward and backward frequencies of this rotor for different values of the number of points (*N*) are calculated and are presented in Table 1. As the Table shows, the presented numerical method was completely convergent, and using *N=11* points, the convergent result was acquired.

**Table 1:** Investigation of the presented analysis convergence.

Forward modes				
*N*	First mode	Second mode	Third mode	Forth mode
5	0.458220	3.438131	5.513690	19.22780
6	3.641992	5.549784	10.61023	18.42175
7	3.690639	19.32481	42.97913	71.73238
8	3.690567	19.55807	44.47682	68.16252
9	3.690486	19.48856	44.95683	69.78575
10	3.690505	19.47988	44.54049	70.35117
11	3.690509	19.48145	44.48862	69.20428
12	3.690509	19.48172	44.50886	69.07583
13	3.690509	19.48170	44.51204	69.15894
14	3.690509	19.48170	44.51141	69.17253
15	3.690509	19.48170	44.51129	69.16765

Backward modes				
*N*	First mode	Second mode	Third mode	Forth mode
5	0.458220	3.401735	5.513690	19.04049
6	3.603335	5.549784	10.61023	18.20216
7	3.651231	19.09245	42.52881	71.15641
8	3.651141	19.32458	44.00614	67.48190
9	3.651052	19.25643	44.49365	69.08069
10	3.651070	19.24777	44.08298	69.67401
11	3.651073	19.24931	44.03078	68.53794
12	3.651074	19.24956	44.05069	68.40716
13	3.651074	19.24955	44.05386	68.48922
14	3.651073	19.24955	44.05324	68.50292
15	3.651074	19.24955	44.05313	68.49812

To check the validity and accuracy of the results, a clamped-free rotor with uniform thickness was considered. The rotor was in the macro dimension *μ =0*without any rotations${\gamma _1}={\gamma _2}=0$. Table 2 demonstrates the first four frequencies for different values *r* and they were compared with the presented values for Euler-Bernoulli beam. According to the Table, for small values of, with a long and thin rotor, the results were extremely similar to those of the Euler-Bernoulli model, confirming the correctness of the study. According to this Table, as the value of the smaller, thicker rotors decreased, the frequency values decreased and were lower than expected by Euler-Bernoulli beam theory. The reason was that, in the Timoshenko beam, when shear deformation was considered, the rotor stiffness decreased while the moment of inertia increased, leading to a decrease in natural frequencies.

**Table 2:** Verification of results against Euler-Bernoulli theory for different slenderness ratios.

	r	$\sqrt {{\lambda _1}}$	$\sqrt {{\lambda _2}}$	$\sqrt {{\lambda _3}}$	$\sqrt {{\lambda _4}}$
	Euler-Bernoulli theory^1^	1.875104	4.694091	7.854757	10.99554
r	0.001	1.875740	4.693990	7.850532	10.98666
	0.01	1.874275	4.679708	7.795132	10.84457
	0.02	1.871802	4.637969	7.639710	10.47199
	0.03	1.867724	4.572589	7.415152	9.984316
	0.05	1.855035	4.391496	6.881894	8.976771

To check the validity of the presented analysis for non-uniform rotors, consider a non-rotating rotor in the macro dimension ${\gamma _1}={\gamma _2}=0,\mu =0,r=0.03$in which the diameter changed according to the relationship given ${d^*}\left( \zeta \right)=1 - 0.5\zeta$. The first four frequencies were calculated and are presented in Table 3 along with the values given by Torabi et al.^33^. The results obtained were close to those reported by Torabi et al.^33^, verifying that the analysis applied here had high accuracy.

**Table 3:** Validity of the presented analysis for a non-uniform rotor.

	$\sqrt {{\lambda _1}}$	$\sqrt {{\lambda _2}}$	$\sqrt {{\lambda _3}}$	$\sqrt {{\lambda _4}}$
Presented analysis	2.142633	4.350976	6.72972	9.038399
Torabi et al^34^.	2.141673	4.347607	6.716715	8.972473

To study the impacts of the rotational V of a nano-rotor around its axial axis${\gamma _1}$, consider a nano-rotor with the following characteristics:

50 $$\begin{array}{*{20}{c}} {{d^*}\left( \zeta \right)=1 - 0.5\zeta }&{,\delta =0.1}&{,r=0.03}&{,\mu =0.1}&{,{\gamma _2}=5} \end{array}$$

To validate the numerical method, we compared the frequencies of a clamped-free rotor with uniform thickness to those predicted by Euler-Bernoulli beam theory.

**Table 4:** Comparison with Euler-Bernoulli Theory^1^.

*r*	Euler-Bernoulli Theory	Numerical Results
1.875	1.875104	1.875740
0.001	4.694091	4.693990
0.01	7.854757	7.850532
0.02	10.99554	10.98666

Table 4 shows that the numerical results for small slenderness ratios (r) closely match those predicted by Euler-Bernoulli theory, confirming the validity of our technique. However, as r decreased, the frequencies predicted by Timoshenko beam theory decreased relative to those from Euler-Bernoulli theory, underscoring the importance of shear deformation and inertia effects.

## Results and discussion

**Fig. 4:**
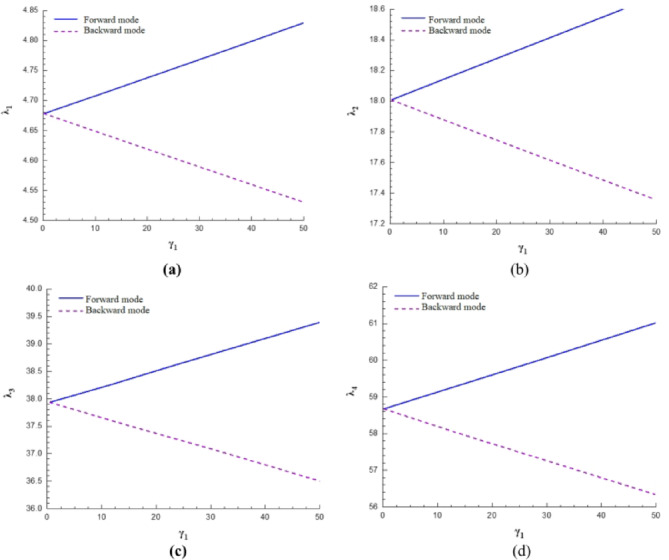
Campbell diagram for the first four frequencies of a nano-rotor.

Due to the geometric symmetry of the nano-rotor and the identical boundary conditions in the transverse directions, the system exhibited vibration in the x and y directions. This resulted in paired modes with identical natural frequencies in the absence of structural asymmetry. Moreover, the coupling between two transverse directions remains symmetric, leading to identical dynamic responses in both planes. Therefore, only one set of representative modes was reported for simplicity, as the corresponding modes in the orthogonal direction followed the same behavior. Figure 4 illustrates the variation of the first four forward and backward frequencies of the nano-rotor as a function of rotational V. In each subplot, the increasing branch corresponded to the forward whirling mode, whereas the decreasing branch represented the backward whirling mode. For a stationary nano-rotor, the forward and backward frequencies were identical; however, due to gyroscopic effects, the forward frequencies increased while the backward frequencies decreased as the rotational speed increased. This trend was consistent with previous studies^28^, confirming the accuracy of the present formulation. In other words, the forward-mode frequencies remained consistently higher than the backward-mode frequencies at non-zero rotational Vs.

The first-quadrant bisector is also illustrated in Fig. 4. Rotational V was equivalent to the natural frequency on this line. These Vs were referred to as crucial Vs because they occurred at the intersection of the Campbell diagram and this bisector, where the nano-rotor’s inherent frequencies and rotational V coincided. The amplitude naturally increased when an external force was applied to a rotor that was revolving at or near its critical V. In other words, the resonance phenomena must be anticipated; therefore, the design should be configured to ensure that the rotational V was sufficiently remote from these locations. To investigate the impact of shaft’s rotational V on nano-rotors, a nano-rotor with the following characteristics should be considered:

51 $$\begin{array}{*{20}{c}} {{d^*}\left( \zeta \right)=1 - 0.25{\zeta ^2}}&{,\delta =0.5}&{,r=0.03}&{,\mu =0.1} \end{array}$$

**Fig. 5:**
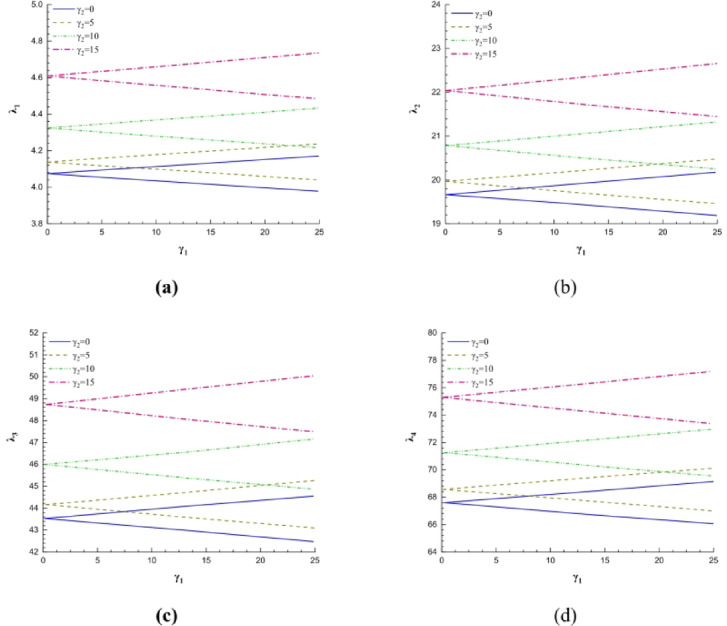
The impacts of the rotational V of the shaft on the Campbell diagram for the first four frequencies of a nano-rotor.

Figure 5 presents the Campbell diagram for the first four vibration frequencies of the nano-rotor at different levels of rotational V applied at one end. The findings show that increasing the rotational V resulted in higher forward and backward frequencies, along with higher corresponding critical Vs. This behavior was primarily attributed to the centrifugal axial force generated along the rotor due to rotation, as described by Eq. (16). The induced axial tension effectively increased the nano-rotor’s structural stiffness, resulting in higher natural frequencies and a shift in the critical points in the Campbell diagram. As the rotational V increased, this centrifugal stiffening effect became more pronounced, leading to a systematic increase in the system’s dynamic stability.

Equation (15) also explains this behavior. According to this relationship, the axial tension force induced by rotation was proportional to V^2^, highlighting its increasing influence at higher Vs. To investigate the effect of shaft’s non-dimensional radius on the behavior of nano-rotors, the following features are considered:

52 $$\begin{array}{*{20}{c}} {{d^*}\left( \zeta \right)=1 - 0.5\zeta }&{,{\gamma _2}=15}&{,r=0.03}&{,\mu =0.1} \end{array}$$

**Fig. 6:**
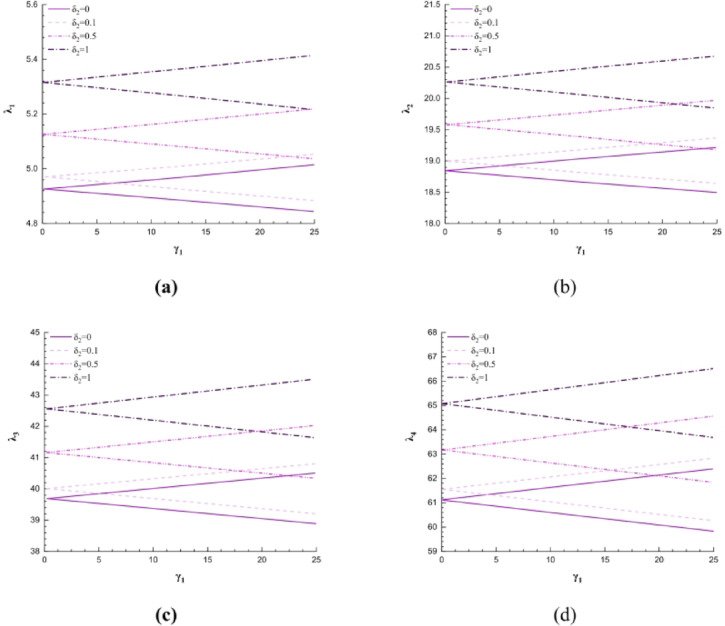
The effects of shaft radius on Campbell diagram for the first four frequencies of nano-rotors.

Figure 6 presents the Campbell diagram for the first four vibration frequencies of the nano-rotor for various values of the non-dimensional radius parameter δ₂. Based on Eq. (25), δ₂ represents the ratio of shaft radius to its length and characterizes the geometric configuration of the nano-rotor. The results show that increasing δ₂ led to higher forward and backward frequencies, along with higher corresponding critical Vs. This behavior was attributed to the increase in centrifugal axial force along the rotor, as described by Eq. (16), where a larger radius enhanced the rotation-induced tension through the term (R + z). Consequently, the effective stiffness of the nano-rotor increased, resulting in an upward shift of frequency branches in the Campbell diagram.

Undoubtedly, the non-local parameter also had a considerable effect on the forward and backward frequencies, Campbell diagram, and the critical Vs. To further investigate this effect, a nano-rotor with the properties defined in Eq. 53 is considered, where the diameter profile, rotational V, and geometric parameters were specified. This configuration enabled a systematic evaluation of the combined impacts of NE and geometric characteristics on the dynamic behavior of the nano-rotor.

53 $$\begin{array}{*{20}{c}} {{d^*}\left( \zeta \right)=\exp \left( { - 0.3\zeta } \right)}&{,{\gamma _2}=15}&{,\delta =0.2}&{,r=0.03} \end{array}$$

**Fig. 7:**
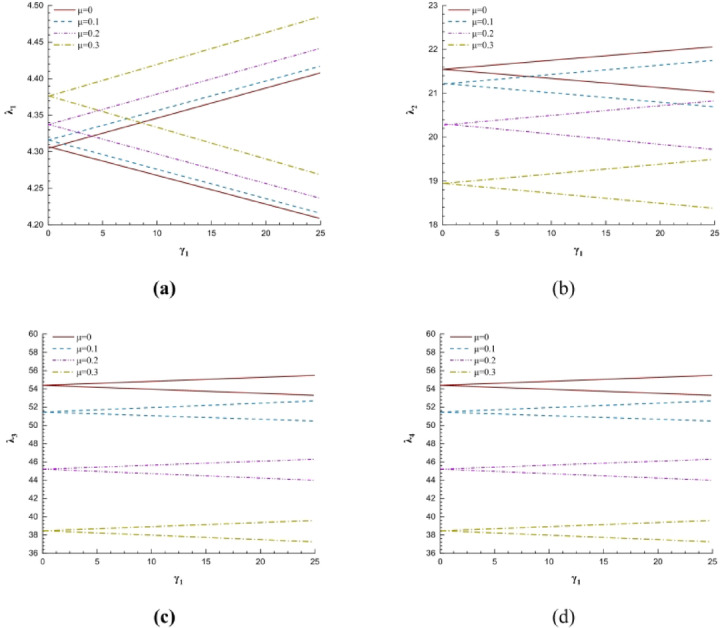
The impacts of non-local parameters on the Campbell diagram for the first four frequencies of a nano-rotor.

The first vibration mode exhibited a distinctly different sensitivity to the non-local parameter compared to the higher modes. As observed in Fig. 7, increasing µ had a negligible effect on the critical V and the forward and backward frequencies of the first mode, whereas in higher modes it resulted in a pronounced decrease in both the natural frequencies and the corresponding critical Vs. This behavior can be rigorously interpreted within the framework of NE theory, in which the stress at a material point depends on the strain field over a finite spatial domain. As the non-local parameter µ increases, the contribution of long-range interatomic interactions becomes more significant, resulting in an effective reduction in structural stiffness and, consequently, a decrease in natural frequencies^34^. Furthermore, higher vibration modes were associated with shorter wavelengths and larger strain gradients, which enhanced their sensitivity to non-local effects. This influence was also reflected in the governing equations and boundary conditions (Eq. 26), where µ appears in higher-order coupled terms. Therefore, the effect of µ is relatively weak in the fundamental mode but becomes increasingly dominant in higher modes.

**Fig. 8:**
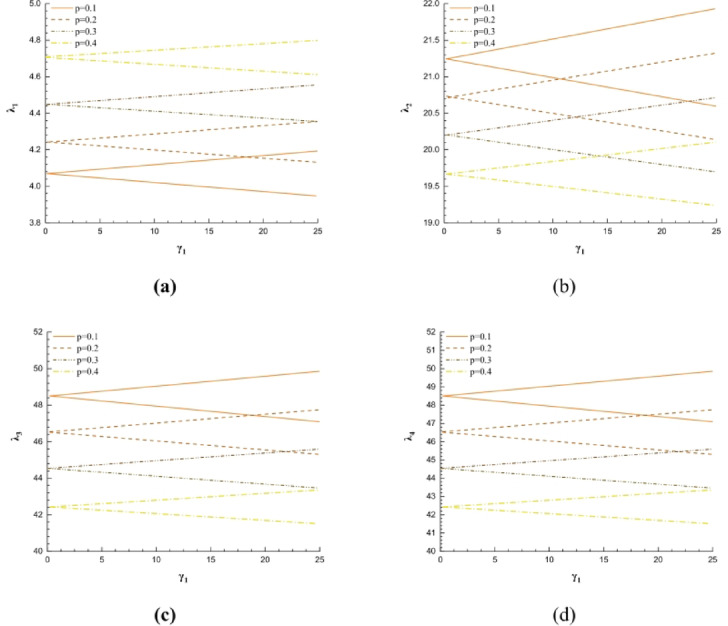
The effects of variation of nano-rotor diameter on Campbell diagram for the first four frequencies of a nano-rotor.

To provide a more rigorous interpretation, the governing Eq. 54 of motion can be expressed in matrix form as:

54 $$M\ddot {u}+G\dot {u}+Ku=0$$

where M, G, and K denote the mass, gyroscopic, and stiffness matrices, respectively. The non-local parameter primarily modified the stiffness matrix via higher-order spatial derivatives, leading to an effective reduction in structural rigidity. In the first vibration mode, the response was dominated by global deformation, and the gyroscopic matrix may partially compensate for the reduced stiffness, resulting in only a minor change in frequency. In contrast, higher modes involved shorter wavelengths and stronger curvature effects, making them more sensitive to the stiffness modification induced by the non-local parameter. Consequently, the decrease in the effective stiffness matrix became more pronounced in higher modes, leading to a noticeable decrease in their corresponding frequencies and critical Vs. In addition to non-local effects, the geometric configuration of the nano-rotor plays a crucial role in determining its dynamic response. In particular, variations in the diameter profile significantly influenced the vibration characteristics. To examine this aspect, a nano-rotor with a variable diameter was considered, defined by the following relationship:

55 $$\begin{array}{*{20}{c}} {{d^*}\left( \zeta \right)=1 - p\zeta {\kern 1pt} {\kern 1pt} {\kern 1pt} {\kern 1pt} {\kern 1pt} {\kern 1pt} {\kern 1pt} {\kern 1pt} {\kern 1pt} {\kern 1pt} ,{\gamma _2}=15}&{,\delta =0.2}&{,r=0.03}&{,\mu =0.1} \end{array}$$

where p denotes the rate of diameter variation along the longitudinal direction. When *p* = 0, the nano-rotor had a uniform cross-section, whereas *p* = 1 corresponded to a fully tapered (conical) configuration. provides information on the effects of Fig. 8 provides information on the effects of backward and forward frequencies and critical Vs on the first four modes. As seen, it was clear that the changes in the first mode were different from other modes because, with an increase in the value of *p* and an increase in the rate of decrease in diameter in the longitudinal direction, the values of mass and stiffness simultaneously decreased. On the one hand, the decrease in mass led to an increase in frequencies. On the other hand, the decrease in stiffness reduced the frequencies, and the result depended on these two effects: an increase in frequency (for the first mode) and a decrease in frequency (from the second mode onward).

Another important parameter that can influence backward and forward frequencies, as well as critical Vs, is the slenderness ratio. According to Eq. (26), an increase in *r* means an increase in slenderness ratio. To study the effects of this variable on the Campbell diagram, consider a rotor with the following characteristics:

56 $$\begin{array}{*{20}{c}} {{d^*}\left( \zeta \right)=1 - 0.3{\zeta ^3}}&{,{\gamma _2}=15}&{,\delta =0.2}&{,\mu =0.1} \end{array}$$

**Fig. 9:**
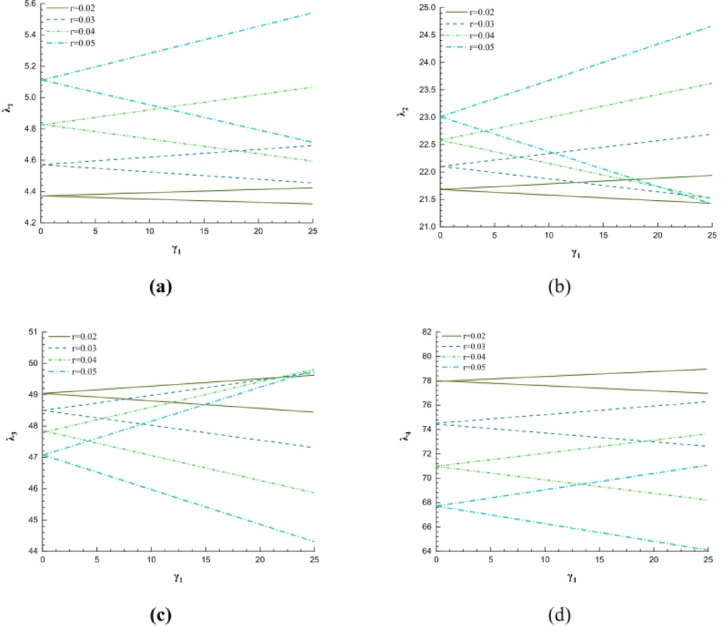
The impacts of the ratio of diameter to length on the Campbell diagram for the first four frequencies.

Figure 9 illustrates the variation of the Campbell diagram for the first four vibration modes of the nano-rotor for various values of the non-dimensional parameter r. It is observed that increasing r leads to an increase in both forward and backward natural frequencies, along with a more pronounced separation between the two branches. Physically, r represents the relative contribution of the cross-sectional moment of inertia to the structural response of the nano-rotor. An increase in r, corresponding to a larger rotor diameter, enhanced the bending rigidity of the structure, thereby increasing the effective stiffness and resulting in higher natural frequencies. In addition, a larger r increased the polar moment of inertia, thereby strengthening the system’s gyroscopic coupling. This enhanced gyroscopic effect amplified the divergence between forward and backward frequencies in the Campbell diagram. Consequently, thicker nano-rotors exhibited both higher vibration frequencies and stronger frequency splitting due to the combined influence of increased structural stiffness and gyroscopic effects.

## Conclusion

This work examined the vibrational characteristics of nano-rotors, emphasizing the impact of rotational motion, structural attributes, and non-local factors on their dynamic behavior. At rest, the anterior and posterior frequencies were equivalent. However, once the rotor began rotation along with its longitudinal axis, this symmetry was disturbed. The forward frequencies increased, whilst the backward frequencies decreased. The frequency divergence intensified with increasing rotational Vs, resulting in higher critical Vs and highlighting the essential role of rotational effects in system stability. In addition to rotational motion, structural factors substantially affected vibrational characteristics. An increase in shaft radius led to higher forward and backward frequencies and higher critical Vs, demonstrating that geometric alterations can be used to optimize rotor dynamics. The work highlighted the intricacy of nanoscale dynamics resulting from non-local strain effects. Increased non-local parameters marginally elevated the forward and backward frequencies in the first vibration mode; however, they led to a significant reduction in higher modes, underscoring the scale-dependent nature of vibrational behavior in nanoscale applications. Moreover, alterations in the rotor’s diameter profile significantly influenced its vibrational response. A more pronounced gradient in diameter reduction along the longitudinal axis led to elevated forward and backward frequencies and critical Vs in the first mode, whereas higher modes showed decreases in frequency with increasing gradient. Similarly, an increased rotor diameter amplified the difference between the forward and backward frequencies, underscoring the essential role of geometric design in vibrational behavior. These discoveries provided essential insights into the dynamics of nanorotors, establishing a foundation for improving their design and performance in advanced nanoscale applications. This work established critical links among rotational V, structural alterations, and non-local effects, facilitating the advancement of high-performance nano-rotors in developing technologies.

## Data Availability

The datasets used and/or analysed during the current study available from the corresponding author on reasonable request.

## List of symbols

u_x_: Transverse displacements in the x direction
u_y_: Transverse displacements in the y-direction
ϕ_x_: Rotations of the cross-section about x
ϕ_y_: Rotations of the cross-section about the y-axis
Ω_1_: Angular velocity of rotation about the transverse axis
Ω_2_: Angular velocity of rotation about the axial direction
γ_1_: Non-dimensional rotational parameter associated with Ω_1_
γ_2_: Non-dimensional rotational parameter associated with Ω_2_
r: Slenderness parameter
δ: Non-dimensional radius parameter
μ: Nonlocal parameter
R: Radius of the nano-rotor
A(z): Cross-sectional area at position ( z )
I(z): Area moment of inertia at position ( z )
I-p: Polar moment of inertia
e_0_a: Non-local length scale parameter
λ: Non-dimensional natural frequency
p: Diameter variation parameter
z: Axial coordinate along the nano-rotor
L: Length of the nano-rotor
